# Risk factors for suicidal ideation among survivors of suicide loss using a psychological support hotline

**DOI:** 10.3389/fpsyt.2025.1513838

**Published:** 2025-02-17

**Authors:** Hong Liang, Zikang Liu, Xingxue Li, Jing An, Liting Zhao, Lin Chen

**Affiliations:** ^1^ Beijing Suicide Research and Prevention Center, Beijing Huilongguan Hospital, Beijing, China; ^2^ World Health Organization Collaborating Center for Research and Training in Suicide Prevention, Beijing, China; ^3^ Department of Psychology, Chengde Medical University, Chengde, China

**Keywords:** psychological support hotline, survivors of suicide loss, suicidal ideation, risk factors, interaction effect

## Abstract

**Introduction:**

Suicide of a loved one significantly affects the physical and mental well-being of close friends and family members, potentially escalating to suicidal ideation in severe cases. Suicidal ideation among survivors of suicide loss is influenced by a multitude of factors. This study aims to investigate the prevalence and risk factors of suicidal ideation among survivors of suicide loss utilizing a psychological support hotline.

**Methods:**

This study included calls identified as survivors of suicide loss from the Beijing Psychological Support Hotline between 2009 and 2023 and evaluated the suicidal ideation of callers. General demographic data and common risk factors of survivors of suicide loss were collected. A comparison was conducted between survivors of suicide loss with and without suicidal ideation. Logistic regression was used to analyze the risk factors for suicidal ideation among survivors of suicide loss. Finally, we evaluated both multiplicative and additive interactions among the risk factors.

**Results:**

Among the 308 calls from survivors of suicide loss, 174 (56.5%) exhibited suicidal ideation. Survivors who had experienced chronic negative life events (P=0.019), severe depression (P =0.010), or high levels of hopelessness (P=0.008) were more likely to develop suicidal ideation. The interaction between depression severity and hopelessness was additive, resulting in a 14.56-fold increase in the risk of developing suicidal ideation when both factors are present compared to their absence.

**Conclusions:**

In the context of psychological support, chronic life events, high levels of hopelessness, and severe depression are risk factors for suicidal ideation among survivors of suicide loss. Moreover, hopelessness and depression demonstrate an additive interaction effect, substantially increasing the risk of suicidal ideation.

## 1 Introduction

Suicide refers to an individual ending their own life as influenced by various psychological factors (1). According to statistics from the World Health Organization, approximately 700,000 individuals die by suicide each year (2). Suicide is a major public health concern in China (3). According to a 2022 report by the National Health Commission of China, the suicide rate in China’s urban populations is 3.59 per 100,000, whereas that in rural populations is 8.25 per 100,000 (4). Approximately 100,000 people die by suicide each year (5). Despite a gradual decline in the suicide rate in China in recent years (6), suicide continues to be a significant cause of mortality in the Chinese population (5).

Suicidal ideation is a critical component of suicidal behavior and is often regarded as the starting point of the suicide process. However, suicidal ideation does not inherently exist within an individual’s cognitive system but rather represents a gradually evolving psychological phenomenon. The emergence and manifestation of suicidal ideation often involve a complex psychological process and dynamic changes (7, 8). The mindsponge mechanism offers a unique perspective for understanding this process, revealing the formation mechanism of suicidal ideation through the lens of multi-layered information filtering (7). This theory allows for the elucidation of how various factors dynamically and continuously interact through pathways such as information input, cognitive filtering, and value adjustment, influencing the emergence and development of an individual’s suicidal ideation.

Suicide has a contagion effect, with each death negatively affecting an average of 6–8 individuals (9). Family members and friends of individuals who die by suicide are classified as survivors of suicide loss. Suicide of an individual has a profound impact on the mental and physical health of their family and friends, imposes significant economic burdens on the household, and can have adverse effects on society, including the risk of copycat suicides (10, 11). Survivors of suicide loss experience a profound level of bereavement that is more intense than that experienced by other grieving individuals. These survivors endure significantly higher levels of psychological distress and face an elevated risk of attempting suicide themselves, categorizing them as a high-risk group for suicidal behavior (12, 13). Research indicates that survivors of suicide loss in China are more likely to develop suicidal tendencies than are other populations (11). The World Health Organization considers interventions for survivors of suicide loss a crucial component of global suicide prevention efforts (14). However, existing research on survivors of suicide loss predominantly centers on Western countries, with a notable lack of studies from China addressing the risk factors associated with suicidal ideation in this population. Consequently, examining the risk factors associated with suicidal ideation among survivors of suicide loss is crucial for advancing suicide prevention strategies.

Under the influence of Chinese cultural norms, suicide is heavily stigmatized in China (15). Individuals fear being labeled as having “psychological problems”. Moreover, traditional culture emphasizes the central role of the family and prioritizes collective interests. Due to the close connections among family members, a single suicide within the family may increase the suicide risk of other members through mechanisms such as emotional resonance and behavioral imitation (16). When faced with the suicide of close family members or friends, survivors frequently employ avoidance and concealment strategies and are reluctant to seek assistance from medical or psychological services (17, 18). Owing to the convenience, expertise, and confidentiality offered by psychological support hotlines (19), individuals are more likely to use these services to mitigate distress. Survivors of suicide loss often require immediate intervention when confronted with the loss of a close friend or family member. The Beijing Psychological Support Hotline is the largest suicide crisis intervention hotline in China, providing interventions and support to tens of thousands of callers annually.

Although existing studies have confirmed that survivors of suicide loss are a high-risk population for suicide, research on the risk factors of suicidal ideation within this group remains relatively limited. Therefore, this study focuses on survivors of suicide loss utilizing psychological helplines, aiming to explore the following two aspects: 1) To investigate the prevalence of suicidal ideation among survivors of suicide loss utilizing psychological support hotlines; 2) To identify the risk factors associated with suicidal ideation among survivors of suicide loss utilizing psychological support hotlines. These findings offer evidence-based support for the provision of intervention services to survivors of suicide loss, thereby establishing a foundation for suicide prevention in this population.

## 2 Materials and methods

### 2.1 Sample

The Beijing Psychological Support Hotline employs a sophisticated assessment system that autonomously generates service tickets for each incoming call (20). Hotline operators collect demographic information and suicide risk factors through interviews, which are then recorded on service tickets. During a call, hotline operators assess the primary issues presented by the caller. The primary issues in the system are categorized into seven main types: family problems, non-family relational issues, work- and study-related or financial difficulties, personal mental health concerns, other adverse events, miscellaneous calls, and requests for further information. Issues related to the suicide of friends or family members are included under other adverse events. If a call is made because of the suicide of a friend or relative, it is classified as a call from a survivor of suicide loss.

This study included all calls received by the Beijing Psychological Support Hotline between January 2009 and December 2023. The exclusion criteria were as follows: 1) call from someone who was not a survivor of suicide loss; 2) calls lasting less than 600 s; 3) silent calls, harassment calls, and hang-up calls; 4) repeat calls; and 5) calls requesting only further information.

This study was approved by the Institutional Review Board of Beijing Huilongguan Hospital (2022-12-Science). Before each call to the psychological support hotline begins, the callers are informed that their conversation will be recorded. The recordings, along with any collected data, were used exclusively for research and educational purposes. The call proceeded only after the caller provided consent.

### 2.2 Measures

Hotline operators evaluated whether a caller identified as a survivor of suicide loss had experienced suicidal ideation in the preceding two weeks by asking, “Have you had any thoughts of suicide during the past two weeks?” If the caller reported no suicidal ideation over the past two weeks, they were classified into the no suicidal ideation group. If the caller reported having experienced suicidal ideation in the past two weeks, they were classified into the suicidal ideation group (21).

Hotline operators assess callers’ depressive emotions using the Depression Diagnostic Screening Scale (22). This scale, which is derived from the nine symptoms of major depressive episodes defined in the DSM-IV, computes the product of the severity and duration of each depressive symptom over the two weeks preceding the call. The aggregate of these products for the nine symptoms forms the total depression score. The total depression score ranges from 0 to 100, with higher scores indicating more severe depressive symptoms. In this study, depression scores were divided into two groups based on the median: a severe depression group (67–100) and a mild depression group (0–66).

Hopelessness and psychological distress were assessed by asking callers the questions “To what degree do you feel hopeful about your future?” and “To what extent do you feel psychological distress?” Scores for hopelessness and psychological distress ranged from 0 to 100, with lower scores indicating higher levels of hopelessness (23). Higher scores for psychological distress caused by the current event reflected a greater level of psychological distress experienced by the callers (23). In this study, the scores for hopelessness and psychological distress were used to define two groups based on the median: a severe hopelessness group (0–40) and a mild hopelessness group (41–100), and a severe distress group (80–100) and a mild distress group (0–79).

The Beijing Psychological Support Hotline has developed standardized methods for assessing common suicide risk factors (20, 24), outlined as follows: History of suicide attempts was assessed by asking, “Have you previously engaged in actions with the intent to end your life?”; the presence of chronic negative life events was assessed by asking, “In the past month, have you experienced any family, work, or other issues that have had a long-term adverse effect on your mental health?”; the presence of acute negative life events was assessed by asking, “In the past week, have there been any events that have had a severe impact on your mental health?”; substance misuse was assessed by asking, “In the past year, have you used sleeping pills, painkillers, anti-anxiety medications, anesthetics, stimulants excessively, casually, or for more than three consecutive months, or have you used any drugs at any time?”; physical illness was assessed by asking, “Do you have any physical disabilities or illnesses that have a severe impact on your daily life?”; history of being maltreated was assessed by asking, “Have you ever experienced physical or sexual abuse?”; and fear of being attacked was assessed by asking, “In the past month, have you been fearful or concerned about being attacked by others?” If the caller responded “yes” to any of the above questions, this indicated the presence of such issues.

This study also gathered data related to bereavement through interviews, specifically focusing on the duration of bereavement and the relationship with the deceased. Additionally, general demographic information was collected, including gender, age, marital status, years of education, work status, history of previous diagnoses, history of previous treatment, and current medication status.

### 2.3 Statistical analysis

Statistical analyses were performed using SPSS version 26.0 and Rx64 version 4.0.3. Chi-squared tests were used to compare the characteristics of survivors of suicide loss with and without suicidal ideation. Binary logistic regression was used to analyze the risk factors for suicidal ideation, with a significance level set at P = 0.05, two-tailed. Multiplicative interaction effects were examined by including the product terms of risk factors in the logistic regression model. The significance level was set at P = 0.05. If the coefficient *B* of the product term in the logistic regression model was not equal to 0 and P < 0.05, this indicated the presence of a multiplicative interaction effect between the two factors.

The epiR package in Rx64 4.0.3 was used to analyze additive interactions, and the ggplot2 package was employed to visualize these interactions. The significance of the additive interactions was assessed using three indicators: RERI, APAB, and SI. If the confidence interval for RERI or APAB did not include 0 and the confidence interval for SI did not include 1, this indicated the presence of an additive interaction between the two factors.

## 3 Results

**Figure 1:**
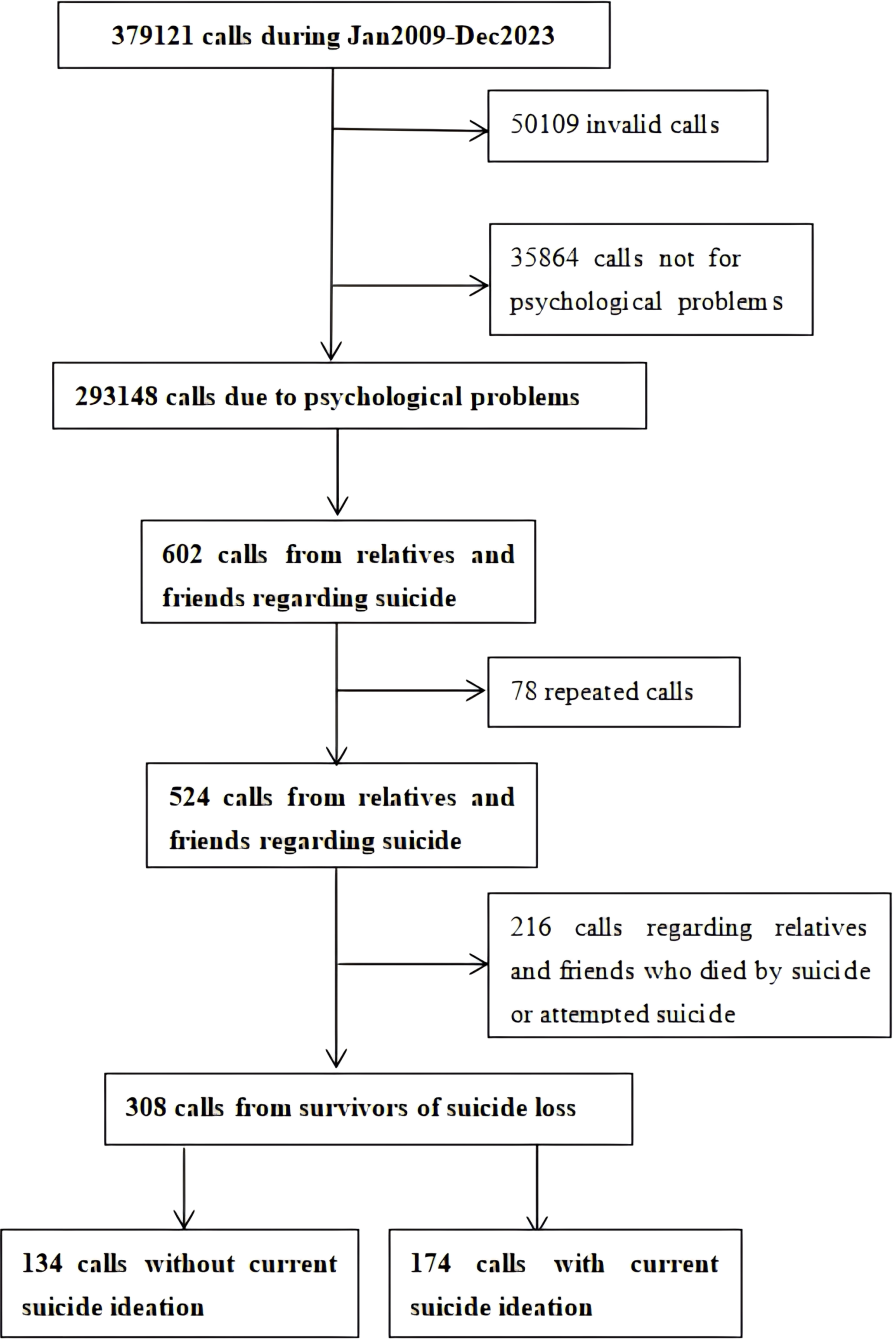
Flowchart of enrolling and screening callers.

The process for selecting survivors of suicide loss is shown in **Figure 1**. A total of 379,121 calls were received between January 2009 and December 2023. After excluding invalid calls, 329,012 valid calls were recorded. After filtering calls that sought only general information, 293,148 calls pertaining to personal issues were recorded. Among the calls related to personal issues, 602 were selected because of problems related to the suicide of friends or relatives. After removing duplicate calls, the data of 524 cases were recorded. After a comprehensive screening process, 308 calls specifically related to suicide by friends or relatives were identified. Among these, 174 callers expressed suicidal ideation (56.5%) and 134 did not (43.5%).

There was no statistically significant difference in suicidal ideation among survivors of suicide loss regarding the duration of bereavement and their relationship with the deceased (**Table 1**). The proportion of survivors of suicide loss with suicidal ideation was higher than those without suicidal ideation among those who were unmarried (63.8% vs 52.2%, P= 0.013), aged 10–18 years (19.5% vs 7.5%, P=0.005), with 0–9 years of education (24.1% vs 11.2%, P=0.001), with a previous diagnosis (29.9% vs 16.4%, P=0.004), and taking psychiatric medication (16.1% vs 7.5%, P= 0.011; see **Table 2**).

**Table 1:** Comparison of bereavement data among survivors of suicide loss with and without suicidal ideation.

Variables	Total (*n* = 308)	Non-suicidal ideation group (*n* = 134)	Suicidal ideation group (*n* = 174)	*χ* ^2^	*P* value
**Duration of bereavement**				4.04	0.257
≤1month	143 (46.4)	70 (52.2)	73 (42.0)		
1–6 months	70 (22.7)	30 (22.4)	40 (23.0)		
7–12 months	28 (9.1)	10 (7.5)	18 (10.3)		
>12 months	67 (21.8)	24 (17.9)	43 (24.7)		
**Relationship with the deceased**				6.03	0.813
Father	37 (12.0)	18 (13.4)	19 (10.9)		
Mother	58 (18.8)	21 (15.7)	37 (21.3)		
Brother	13 (4.2)	6 (4.5)	7 (4.0)		
Sister	13 (4.2)	5 (3.7)	8 (4.6)		
Son	16 (5.2)	10 (7.5)	6 (3.4)		
Daughter	11 (3.6)	6 (4.5)	5 (2.9)		
Partner	40 (13.0)	16 (11.9)	24 (13.8)		
Spouse	25 (8.1)	9 (6.7)	16 (9.2)		
Friend	53 (17.2)	23 (17.2)	30 (17.2)		
Classmate	12 (3.9)	5 (3.7)	7 (4.0)		
Other relatives	30 (9.7)	15 (11.2)	15 (8.6)		

**Table 2:** Comparison of demographic data among survivors of suicide loss with and without suicidal ideation.

Variables	Total (*n* = 308)	Non-suicidal ideation group (*n* = 134)	Suicidal ideation group (*n* = 174)	*χ* ^2^	*P* value
**Gender**				0.27	0.603
Female	188 (61.0)	84 (62.7)	104 (59.8)		
Male	120 (39.0)	50 (37.3)	70 (40.2)		
**Marital Status**				8.64	0.013
Unmarried	181 (58.8)	70 (52.2)	111 (63.8)		
Married	95 (30.8)	53 (39.6)	42 (24.1)		
Divorced	32 (10.4)	11 (8.2)	21 (12.1)		
**Work status**				5.11	0.078
Student	78 (25.3)	31 (23.1)	47 (27.0)		
Employed	155 (50.3)	77 (57.5)	78 (44.8)		
Unemployed	72 (23.4)	25 (18.7)	47 (27.0)		
**Age (years)**				12.86	0.005
10–18	44 (14.3)	10 (7.5)	34 (19.5)		
19–25	84 (27.3)	33 (24.6)	51 (29.3)		
26–49	157 (51.0)	79 (59.0)	78 (44.8)		
50–78	21 (6.8)	12 (9.0)	9 (5.2)		
**Education (years)**				13.69	0.001
≤9	57 (18.5)	15 (11.2)	42 (24.1)		
10–16	224 (72.7)	103 (76.9)	121 (69.5)		
>16	23 (7.5)	16 (11.9)	7 (4.0)		
**History of previous treatments**				6.75	0.080
Untreated	175 (56.8)	86 (64.2)	89 (51.1)		
Outpatient	87 (28.2)	30 (22.4)	57 (32.8)		
Psychological counseling	23 (7.5)	12 (9.0)	11 (6.3)		
Other methods	16 (5.2)	5 (3.7)	11 (6.3)		
**Previous diagnosis history**				8.32	0.004
No history of previous diagnoses	227 (73.7)	111 (82.8)	116 (66.7)		
History of previous diagnoses	74 (24.0)	22 (16.4)	52 (29.9)		
**Current medication status**				6.48	0.011
Not taking psychotropic medications	265 (86.0)	128 (95.5)	137 (78.7)		
Taking psychotropic medications	38 (12.3)	10 (7.5)	28 (16.1)		

Because of missing data, for most variables, percentages do not total 100%.

The proportion of survivors of suicide loss with suicidal ideation was significantly higher among those who reported chronic life events (62.1% vs 43.3%, P < 0.001), fear of being attacked (17.2% vs 7.5%, P=0.008), history of being maltreated (14.4% vs 6.0%, P=0.013), history of suicide attempts (28.2% vs 10.4%, P < 0.001), severe psychological distress (67.2% vs 45.5%, P < 0.001), severe hopelessness (54.0% vs 21.6%, P < 0.001), and severe depression (55.7% vs 23.1%, P < 0.001; see **Table 3**).

**Table 3:** Comparison of suicide-related factors among survivors of suicide loss with and without suicidal ideation.

Variables	Total (*n* = 308)	Non-suicidal ideation group (*n* = 134)	Suicidal ideation group (*n* = 174)	*χ* ^2^	*P* value
**Chronic life events**	166 (53.9)	58 (43.3)	108 (62.1)	16.37	<0.001
**Acute life events**	128 (41.6)	57 (42.5)	71 (40.8)	0.03	0.875
**Fear of being attacked**	40 (13.0)	10 (7.5)	30 (17.2)	7.13	0.008
**History of being maltreated**	33 (10.7)	8 (6.0)	25 (14.4)	6.15	0.013
**Physical illness**	27 (8.8)	11 (8.2)	16 (9.2)	0.13	0.714
**Substance misuse**	20 (6.5)	6 (4.5)	14 (8.0)	1.70	0.193
**History of suicide attempts**	63 (20.5)	14 (10.4)	49 (28.2)	15.20	<0.001
**Hopelessness score**				34.18	<0.001
Mild	139 (45.1)	83 (59.7)	56 (40.3)		
Severe	123 (39.9)	29 (21.6)	94 (54.0)		
**Psychological distress score**				16.66	<0.001
Mild	97 (31.5)	58 (59.8)	39 (40.2)		
Severe	178 (57.8)	61 (45.5)	117 (67.2)		
**Severity of depression score**				38.40	<0.001
Mild	124 (40.3)	78 (62.9)	46 (37.1)		
Severe	128 (41.6)	31 (23.1)	97 (55.7)		

Because of missing data, for most variables, percentages do not total 100%.

After adjusting for demographic variables, increased risk of suicidal ideation among survivors of suicide loss was associated with chronic life events, severe hopelessness, and severe depression (**Table 4**). **Table 5** shows that Models 1, 2, and 3 included multiplicative interaction effects between the two factors, which did not reach statistical significance.

**Table 4:** Logistic regression analysis of risk factors for suicidal ideation among survivors of suicide loss.

Variables	B	B-SD	*Wald*	*P*	*Or*	OR 95% CI	
						Lower limit	Upper limit
**Female**	0.14	0.39	0.13	0.715	1.15	0.54	2.47
**Marital Status**							
Unmarried			1.55	0.460			
Married	0.29	0.55	0.28	0.595	1.34	0.45	3.96
Divorced	0.84	0.69	1.49	0.222	2.31	0.60	8.85
**Age (years)**							
10–18			4.69	0.196			
19–25	-0.27	0.64	0.18	0.671	0.76	0.22	2.69
26–49	-1.23	0.72	2.87	0.091	0.29	0.07	1.21
50–78	-2.06	1.07	3.71	0.054	0.13	0.02	1.04
**Education (years)**							
≤9			5.06	0.080			
10–16	-0.58	0.49	1.42	0.233	0.56	0.22	1.45
>16	-1.79	0.80	5.05	0.025*	0.17	0.04	0.80
**On psychotropic medications**	0.71	0.69	1.06	0.302	2.04	0.53	7.90
**History of previous diagnoses**	0.25	0.55	0.21	0.651	1.28	0.44	3.79
**History of suicide attempts**	0.49	0.47	1.10	0.293	1.64	0.65	4.09
**History of being maltreated**	0.52	0.63	0.68	0.409	1.69	0.49	5.85
**Fear of being attacked**	0.13	0.51	0.07	0.798	1.14	0.42	3.10
**Chronic life events**	0.95	0.40	5.50	0.019*	2.57	1.17	5.67
**Severe psychological distress**	0.25	0.39	0.41	0.525	1.28	0.60	2.72
**Severe hopelessness**	1.05	0.40	6.94	0.008**	2.86	1.31	6.25
**Severe depression**	0.97	0.38	6.69	0.010*	2.64	1.27	5.52

*P<0.05, **P<0.01.

**Table 5:** Multiplicative interaction effects of risk factors for suicidal ideation among survivors of suicide loss.

Model	Variables	B	B-SD	*Wald*	*P*	*Or*	OR 95% CI	
							Lower limit	Upper limit
**Model 1**	Chronic life events	0.72	0.38	3.60	0.058	2.06	0.98	4.36
	Hopelessness	1.40	0.56	6.26	0.012	4.07	1.36	12.23
	Chronic life events × Hopelessness	0.15	0.67	0.05	0.818	1.17	0.33	4.34
**Model 2**	Chronic life events	1.14	0.40	8.13	0.004	3.12	1.43	6.82
	Depression	2.00	0.51	15.23	<0.001	7.35	2.70	20.02
	Chronic life events × Depression	-0.71	0.62	1.33	0.249	0.49	0.15	1.64
**Model 3**	Hopelessness	1.12	0.45	6.36	0.012	3.07	1.28	7.34
	Depression	1.12	0.39	8.28	0.004	3.05	1.43	6.53
	Hopelessness × Depression	0.44	0.63	0.48	0.487	1.55	0.45	5.39

**Figure 2:**
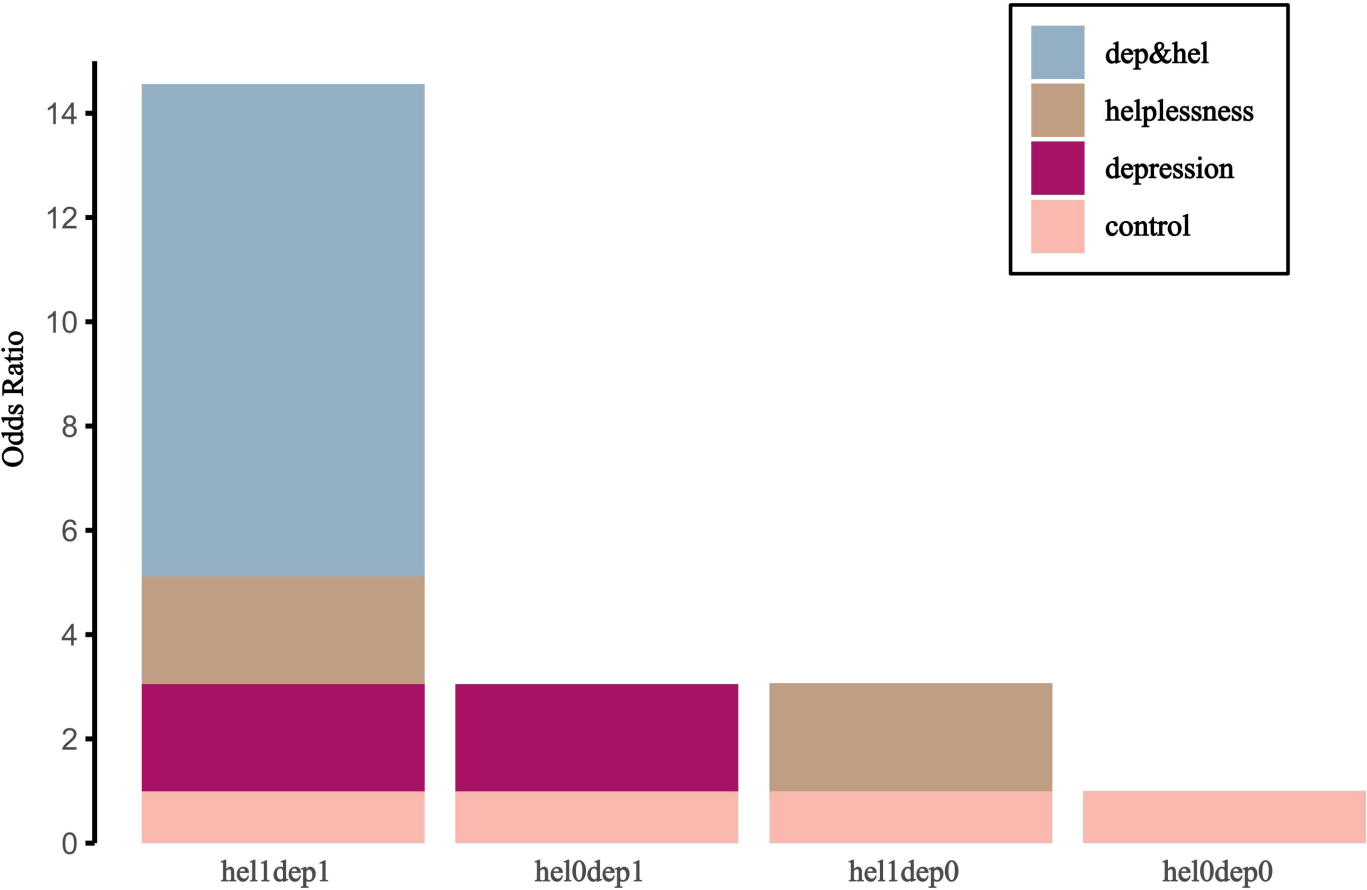
Additive interaction effects of risk factors for suicidal ideation among survivors of suicide loss. dep, depression; hel, helplessness; hel0, mild hopelessness; hel1, severe hopelessness; dep0, mild depression; dep1, severe depression.

Interaction assessment metrics indicated a significant additive interaction between depression severity and hopelessness. The confidence interval for RERI or APAB did not include 0, and the confidence interval for SI did not include 1. Of the combined risk associated with depression severity and hopelessness, 65% could be attributed to their interaction. Furthermore, individuals exposed to both hopelessness and depression exhibited a 14.56-fold increase in the risk of suicidal ideation compared to those unexposed to either factor (see **Table 6**). **Figure 2** presents a visual analysis of the additive interactions.

**Table 6:** Evaluation metrics of additive interaction effects of risk factors for suicidal ideation among survivors of suicide loss.

Variables		OR (95%CI)	RERI (95%CI)	APAB (95%CI)	SI (95%CI)
**Chronic life events**	**Hopelessness**		4.66 (-2.30–11.63)	0.48 (-0.02–0.97)	2.13 (0.68–6.69)
**No**	Mild hopelessness	1.00			
	Severe hopelessness	2.06 (0.98–4.36)			
**Yes**	Mild hopelessness	4.07 (1.36–12.23)			
	Severe hopelessness	9.80 (4.40–21.80)			
**Chronic life events**	**Depression**		1.81 (-6.23–9.84)	0.16 (-0.50–0.83)	1.21 (0.50–2.93)
**No**	Mild depression	1.00			
	Severe depression	3.12 (1.43–6.81)			
**Yes**	Mild depression	7.35 (2.70–20.02)			
	Severe depression	11.28 (5.16–24.63)			
**Hopelessness**	**Depression**		9.43 (-1.50–20.37)	0.65 (0.34–0.96)	3.29 (1.19–9.07)
**Mild**	Mild depression	1.00			
	Severe depression	3.05 (1.43–6.53)			
**Severe**	Mild depression	3.07 (1.28–7.34)			
	Severe depression	14.56 (6.37–33.29)			

## 4 Discussion

This study showed that the prevalence of suicidal ideation among survivors of suicide loss using the psychological support hotline was 56.5%. This rate is lower than that found among adolescents (25) and individuals with mental disorders (26) who use the psychological support hotline but higher than the rate observed among repeat callers (27) and those with general concerns (24). In rural China, the rate of detection of suicidal ideation among survivors of suicide loss is 31.5% (5). The higher detection rate of suicidal ideation among survivors of suicide loss who made use of the psychological support hotline compared to other samples may be attributed to the fact that these survivors are drawn from helpline users and are all actively seeking help, unlike other groups. The psychological support hotline provides an anonymous environment, effectively reducing the stigma associated with suicide (28), thereby encouraging individuals to disclose suicidal ideation more openly. However, the mindsponge mechanism explains the emergence and development of suicidal ideation among survivors of suicide loss. According to the mindsponge mechanism, the core value system of survivors of suicide loss is often profoundly disrupted by the suicide of their loved ones (7). This event not only inflicts emotional trauma on survivors of suicide loss but may also lead to significant alterations in their self-perception and profound disruptions in their sense of life’s meaning. The collapse of the core value system makes survivors of suicide loss more susceptible to negative emotions, including feelings of helplessness and despair. Furthermore, survivors of suicide loss are continuously exposed to information related to their loved one’s suicide, such as societal stigmatization of suicide, external accusations directed at survivors, and the specific circumstances and details surrounding the suicide. These external pieces of information continuously flow into the cognitive system of survivors of suicide loss through the peripheral information pool (7). During this process, the cognitive filters of survivors of suicide loss evaluate and select the incoming information. If individuals tend to focus on negative information while rejecting or ignoring positive support and affirmative messages, these negative inputs may further reinforce their sense of helplessness (8). This tendency of information selection not only exacerbates the cognitive dissonance of survivors of suicide loss but also hinders their ability to restructure their collapsed core value system, trapping them in a vicious cycle of negative cognition. Ultimately, under the accumulation of emotional suffering and cognitive imbalance, survivors of suicide loss may perceive suicide as a means to alleviate psychological pain or restore inner balance, leading to the development of intense suicidal ideation.

In this study, suicidal ideation among survivors of suicide loss was not statistically associated with their relationship to the deceased, consistent with findings from other studies (29). Among bereaved individuals experiencing accidental or natural deaths, the relationship to the deceased shows no significant association (30). However, the level of intimacy between the deceased and the bereaved significantly impacts the latter’s physical and mental health (31). For survivors of suicide loss, the level of intimacy with the deceased does not have a significant impact on their mental health or suicidal ideation. This may be because individuals with suicidal tendencies often struggle to form deep, intimate relationships, resulting in less closeness between survivors of suicide loss and the deceased compared to other types of bereaved individuals (32). The results of this study indicate that the time since bereavement does not have a significant impact on suicidal ideation among survivors of suicide loss. Although studies suggest that suicidal ideation among survivors of suicide loss may decrease over time (33). However, in the context of Eastern cultures, traditional cultural factors may diminish the mitigating effect of time on suicidal ideation among survivors of suicide loss. For instance, the emphasis on family and kinship in traditional cultures often causes survivors of suicide loss to experience prolonged guilt and self-blame for failing to save their loved one’s life. Meanwhile, cultural norms emphasizing stoicism and self-restraint may hinder survivors of suicide loss from expressing their grief, thereby exacerbating their psychological distress (34). Overall, these cultural factors may sustain the emotional suffering of survivors of suicide loss, attenuating the natural healing effect of time on alleviating suicidal ideation, and in some cases, may even exacerbate the deterioration of their mental health.

Numerous studies of psychological support hotlines have identified chronic life events as significant risk factors that intensify suicidal ideation among callers (25). In this study, we reached the same conclusion, further supporting the impact of chronic life events on suicidal ideation among survivors of suicide loss. Furthermore, bereavement, as an acute negative life event, has been widely demonstrated to significantly increase the risk of suicide (35). Compared to the acute impact of bereavement, chronic life events are characterized by the long-term accumulation of stress, gradually depleting an individual’s psychological resources and coping capacity (36). When stress exceeds an individual’s tolerance threshold, it may trigger intense feelings of hopelessness and helplessness, ultimately leading to the emergence of suicidal ideation (37, 38). In the context of eastern cultures, approaches to coping with chronic life events differ from those in western countries (39). Particularly in situations where suicide bereavement triggers other chronic life events, the strong emphasis on familial responsibility and ethics in eastern cultures often intensifies the sense of responsibility felt by survivors of suicide loss (40). This cultural pressure imposes a more complex emotional burden and social stress on them when dealing with additional chronic life events, thereby impacting the mental health of survivors of suicide loss. These cultural characteristics not only intensify the psychological burden of survivors of suicide loss but also further weaken their coping capacity, thereby accelerating the onset of crises. Psychological interventions for this group should address the short-term impacts of bereavement while identifying and alleviating stressors associated with chronic life events.

In this study, a high level of depression was identified as a risk factor for suicidal ideation among survivors of suicide loss, which is consistent with the findings of other studies (21). Previous studies have demonstrated a significant positive correlation between depressive mood and suicidal ideation, with depressive mood identified as a predictor of suicidal ideation (41). Additionally, survivors of suicide loss often experience complicated grief, a psychological state that exacerbates their level of depression and intensifies suicidal ideation (42, 43). The impact of bereavement on survivors of suicide loss extends beyond the event itself, reaching into deeper emotional dimensions. Bereavement often generates new life stressors, further intensifying emotional distress, triggering or exacerbating depressive symptoms, and thereby increasing the intensity of suicidal ideation (44). In future helpline operations, emphasis should be placed on the assessment and intervention of depressive states among survivors of suicide loss. Building social support networks to reduce social isolation may help alleviate depressive symptoms, thereby preventing the emergence of suicidal ideation (45).

Hopelessness is a significant risk factor for suicidal ideation (21), which is consistent with the findings of this study. High levels of hopelessness can exacerbate suicidal ideation among survivors. Hopelessness is often characterized by a pessimistic outlook on the future and a perceived loss of problem-solving ability. It is not only a significant indicator of psychological distress but also a critical predictor of suicidal behavior (46). In studies related to psychological support hotlines, hopelessness has also been confirmed as a significant risk factor for suicidal ideation across various groups. For example, High levels of helplessness, recognized as a significant risk factor, can exacerbate suicidal ideation among adolescents, adult women, and individuals grappling with gambling issues who seek help through psychological support hotlines (25, 47, 48). These studies indicate that hopelessness exhibits a cross-cultural and universal predictive effect across various populations. For survivors of suicide loss, high levels of hopelessness not only directly intensify suicidal ideation but also further deteriorate mental health by affecting their emotional state (49). For example, hopelessness may amplify feelings of loneliness, guilt, and anger, making it more challenging for survivors of suicide loss to recover from bereavement. Furthermore, hopelessness may lead to complex grief reactions. If left unaddressed, these reactions can develop into prolonged grief disorder, causing lasting harm to the physical and mental health of survivors of suicide loss (50).

The analysis in this study indicated that there was an additive interaction between the level of depression and helplessness in relation to suicidal ideation among survivors of suicide loss. However, a literature review reveals that studies on the interaction between risk factors for suicidal ideation among survivors of suicide loss remain limited. This underscores the necessity of this study, providing a novel perspective for exploring this field. According to the findings of this study, survivors of suicide loss experiencing both depression and helplessness exhibit significantly higher levels of suicidal ideation compared to those with either condition alone. This result suggests a synergistic effect between these two risk factors, indicating that the risk of suicidal ideation significantly increases when individuals simultaneously experience depression and helplessness. This additive effect may stem from depression exacerbating individuals’ negative cognition, while helplessness further depletes their coping resources and psychological resilience, making them more prone to a state of despair (51).

This study has several limitations. First, owing to the limitations of the hotline system in data collection, the data gathered in this study were not comprehensive. The study did not involve extensive interviews with survivors of suicide loss, but was solely based on the fixed evaluation system of the hotline. Second, this study was cross-sectional and did not include a longitudinal follow-up of survivors of suicide loss, which limits our understanding of how psychological health status changes over time. Third, although we collected data from survivors of suicide loss who called the hotline between 2009 and 2023, the sample size was relatively small. Finally, The data collection relies on self-reports from callers, which may affect data accuracy. In future research, the sample size can be further expanded to include more survivors of suicide loss from diverse backgrounds, enriching bereavement-related data such as specific causes of death and detailed contexts of bereavement. Additionally, efforts can be made to quantify eastern cultural factors and incorporate them into models to comprehensively examine their potential impact on the suicidal ideation of survivors of suicide loss. In terms of analytical methods (52), the bayesian mindsponge framework can be utilized to delve into the complex psychological mechanisms underlying the suicidal ideation of survivors of suicide loss, including the interactions among cultural, cognitive, emotional, and social support factors.

## 5 Conclusions

This study, based on data from survivors of suicide loss in psychological support hotlines samples, systematically explored the risk factors for suicidal ideation in this population. The results indicate that chronic life events, high levels of depression, and heightened hopelessness are significant risk factors for suicidal ideation among survivors of suicide loss. Furthermore, the study identified a significant additive interaction between the levels of depression and hopelessness. This suggests that when survivors of suicide loss experience both severe depressive symptoms and intense hopelessness, the risk of suicidal ideation is significantly higher than the sum of these factors acting independently. The findings of this study provide important insights for psychological interventions targeting survivors of suicide loss. Future intervention practices should focus on the chronic life events, hopelessness, and depression levels experienced by survivors of suicide loss. Timely assessment of potential suicide risks, early identification of problems, and implementation of effective early intervention strategies. These efforts can reduce suicidal ideation among survivors of suicide loss, lower the risk of suicidal behaviors, and ultimately enhance their overall mental health.

## Data Availability

The raw data supporting the conclusions of this article will be made available by the authors, without undue reservation.
